# Chloride of Soda in Cancerous Ulcers

**Published:** 1876-07

**Authors:** G. M. Rivers


### CHLORIDE OF SODA IN CANCEROUS ULCERS.

By G. M. RIVERS, M.D.

About three years ago, I was requested to visit an old lady with an ugly ulcer of the left breast. The whole gland was involved in the disease, hard and uneven to the touch—the ulcer three inches in diameter, quite deep, with raised, ragged and irregular edges; in fact, presenting all the appearance of true cancer. I had previously attended two cases of cancer of the breast, both of which presented the same appearance and proved fatal. I held out no hope of recovery, but advised and instituted, as I believed, a purely palliative treatment. I directed the ulcer to be covered with simple cerate, and, to destroy the odor, to be washed twice a day with a solution of chloride of soda—Laberaque's solution, in proportion of three ounces of the chloride to five of water. In three or four months, with no other treatment whatever, except small doses of morphine to relieve pain, the ulcer had perfectly healed, the swelling and inflammation subsided, and the patient cured. It has now been more than two years, and the disease shows no disposition to return.

The second case upon which I tried this remedy was a young negro woman, about twenty, with half the left nostril and a large portion of the upper lip destroyed. This woman had been to the hospital in Charleston, and the physicians there had pronounced it cancer. Upon this case I used the chloride very freely, injecting into the nares as well as applying it externally. In four months, the patient was cured, the last integument restored, leaving less deformity than I thought possible.* In this case, also, I used no other remedy, except to protect it from the air with simple cerate.

The question will naturally occur, Were these cases true cancers? Their recovery alone makes me doubtful. I certainly had no doubts when I first saw them. But may not the chloride of soda be a remedy? It is as good as anything else. Why not try it? Some of our most useful remedies have been discovered by accident.

*This woman's father died with cancer.

